# A Quantitative Study of the Secondary Acoustic Radiation Force on Biological Cells during Acoustophoresis

**DOI:** 10.3390/mi11020152

**Published:** 2020-01-30

**Authors:** Davood Saeidi, Mohsen Saghafian, Shaghayegh Haghjooy Javanmard, Martin Wiklund

**Affiliations:** 1 Department of Mechanical Engineering, Isfahan University of Technology, Isfahan 84156-83111, Iran; davoudsae@gmail.com (D.S.); saghafian@cc.iut.ac.ir (M.S.); 2 Department of Applied Physics, Royal Institute of Technology, KTH-AlbaNova, SE-106 91 Stockholm, Sweden; 3 Department of Physiology, Applied Physiology Research Center, Cardiovascular Research Institute, Isfahan University of Medical Sciences, Isfahan 81745, Iran; sh_haghjoo@med.mui.ac.ir

**Keywords:** acoustophoresis, secondary acoustic radiation forces, cell manipulation

## Abstract

We investigate cell-particle secondary acoustic radiation forces in a plain ultrasonic standing wave field inside a microfluidic channel. The effect of secondary acoustic radiation forces on biological cells is measured in a location between a pressure node and a pressure anti-node and the result is compared with theory by considering both compressibility and density dependent effects. The secondary acoustic force between motile red blood cells (RBCs) and MCF-7 cells and fixed 20 µm silica beads is investigated in a half-wavelength wide microchannel actuated at 2 MHz ultrasonic frequency. Our study shows that the secondary acoustic force between cells in acoustofluidic devices could play an important role for cell separation, sorting, and trapping purposes. Our results also demonstrate the possibility to isolate individual cells at trapping positions provided by silica beads immobilized and adhered to the microchannel bottom. We conclude that during certain experimental conditions, the secondary acoustic force acting on biological cells can dominate over the primary acoustic radiation force, which could open up for new microscale acoustofluidic methods.

## 1. Introduction

Particle and cell manipulation by utilizing the acoustic radiation force has been extensively investigated [1,2,3,4,5,6,7]. The source of this manipulation is the sound wave scattered from suspended objects with different acoustic properties relative to surrounding medium. When applied to microfluidic channels, the method is often referred to as acoustophoresis [8]. Although the primary acoustic radiation force is the most important force in acoustophoresis, acoustic streaming [9,10,11,12,13], and the secondary radiation force also play an important role at specific experimental conditions. On the one hand, the primary acoustic radiation force acts on single particles and has been widely investigated and is today well understood [14]. On the other hand, the secondary acoustic radiation force acts between two or several particles and has not been studied as extensively, especially in the case of different particles interacting such as cells interacting with solid particles. The secondary acoustic force typically becomes important when there are two or more bubbles or particles in close proximity to each other [15] and causes attractive or repulsive forces. Although this phenomenon has been widely studied theoretically and experimentally on bubble pairs by, for example, Bjerknes [16], Crum [17], and Doinikov [18], there are fewer studies focusing on the interaction between solid particles in acoustophoresis. In particular, there are few existing experimental studies on such solid particle interaction. Zheng and Apfel [19] found that the magnitude and direction of the secondary acoustic force between two objects in acoustic field depend strongly on the relative orientation of the particle pair to the wave propagation direction. In another study, Garcia-Sabate et al. [20] presented a new experimental measurement method for calculating the secondary acoustic force between two neighboring particles in an acoustic field. Their study was limited to the case when two solid particles are already located within the pressure nodal line and hence without any primary acoustic force. Thus, at this condition the secondary acoustic force is the only acting force on the particle.

By considering particles much smaller than the wavelength, the effect of the secondary acoustic radiation force can be divided into two terms, the monopole and the dipole terms. In a theoretical study, Gröschl [15] presented a formula which includes these two terms based on the particle positions relative to the pressure nodal line. In the following, Silva and Bruus [21] introduced a theoretical expression for acoustic interparticle forces between small spherical suspended particles in an ideal fluid when they are close to each other. In their study both compressible liquid droplets and elastic microspheres were considered in the Rayleigh limit (when the particle size is much smaller than the acoustic wavelength). Their results showed that when two particles are in the vicinity of each other there is an area in which particles show attraction or repulsion to each other in the perpendicular direction to the wave propagation direction. In 2015, Sepehrirahnama et al. [22] presented a numerical scheme to calculate the secondary acoustic force in an ideal fluid. Their method is based on isothermal theory and Helmholtz equation with proper boundary conditions in order to find the force acting on the particles. In 2017, Baasch et al. [23] implemented an algorithm of displacement level to model the complete trajectory of particles while considering the particle–particle contact, as well as hydrodynamic and acoustic interaction on particles. They compared their simulation with experimental data which had a good agreement in particle trajectory. In 2017, Wang et al. [24] studied the effects of unsteady inertial forces on the particle trajectory while they considered the particle–particle interaction in an acoustic standing wave field. They showed that unsteady inertial forces such as hydrodynamic interaction can reduce the particle transversal displacement. The secondary acoustic radiation force was also investigated numerically by Habibi et al. [25] for large diameter particles relative to the acoustic wavelength. They showed that solid sphere resonance frequencies have a remarkable impact on the secondary acoustic force and lead to limited narrow frequency bandwidth in patterning large particles. In a recent study Mohapatra et. al. [26] investigated the secondary acoustic force for three different sizes of polystyrene beads and compared their results with theoretical values. Although their results were in the same order of magnitude with theoretical values, there were remarkable differences in some cases. Their study was limited to the case where two rigid particles were approaching the pressure nodal line. In summary, what is still missing in a majority of reported experimental studies is an investigation of the influence of monopole and dipole effects, as defined in the Gröschl model [15]. Since these effects are related to acoustic pressure and velocity, respectively, such studies need to consider secondary acoustic radiation forces in arbitrary positions in a standing wave.

In a previous study by the authors [27], we presented a new method to experimentally measure the secondary acoustic force between polystyrene particles in an arbitrary position in a one-dimensional standing wave. We showed that secondary acoustic forces can cause significant deflections of particle trajectories when particles are in close proximity of each other. In this study, we use a similar approach to study the secondary acoustic force between biological cells and silica particles, and we discuss how this behavior of cells in the acoustic field can affect the purity of separated or manipulated particles and cells. In addition, we show that the secondary acoustic radiation force can be used in a new acoustic trapping method for biological cells in microfluidic systems. Our results can also be used for optimizing cell and particle concentrations in acoustofluidic separation systems by minimizing the secondary acoustic force undesired effects. Furthermore, the measured secondary acoustic force is compared with an extension of the Gröschl [15] theory by considering both monopole and dipole effects, as well as particles with different material properties. For the purpose of achieving significant secondary acoustic forces, we used 20 µm silica particles immobilized at fixed positions in the microfluidic channel containing a cell suspension.

## 2. Interparticle Forces in Acoustophoresis

### 2.1. Acoustic Forces

On the basis of the Gorkov theory Equation (2), exposing a particle in an acoustic standing wave, causes a time-averaged force on the particle surface known as the primary acoustic force, $F_{\mathrm{pr}}$. This force in a one-dimensional acoustic wave in an ideal fluid can be expressed as:

(1) $$F_{\mathrm{pr}}\left(y\right)=4\pi r^{3}E_{\mathrm{ac}}k\;\phi \;\sin\left(2ky\right)$$

(2) $$\phi =1-\frac{\kappa_{p}}{\kappa_{f}}+\frac{3\left(\rho_{p}-\rho_{f}\right)}{2\rho_{p}+\rho_{f}}$$

Here, $E_{\mathrm{ac}}$, ϕ, and k are the acoustic energy density, acoustic contrast factor, and wave number, respectively, and y is the distance from the first pressure node. For the application of this theory to a half-wavelength wide microchannel with a pressure node oriented along the channel direction, *y* is the horizontal direction perpendicular to the channel direction. Furthermore, ρ and κ are the density and compressibility while subscripts f and p refer to the fluid and the particle, respectively.

In such a case when there is a small distance between the particles or there is a high concentration of cells and particles during the separation process in an acoustic field, an additional force called the secondary acoustic radiation force can become significant between particles or cells. This force contains two different effects caused by compressibility and density variations between particles or cells and the surrounding medium. Apfel [28] studied bubbles and derived the secondary acoustic radiation force, $F_{\mathrm{se}}$, based on the compressibility effect only. Although the compressibility-based effect of secondary acoustic force plays an influential role in the interaction force between bubbles, the density effect of the secondary acoustic radiation force is dominant in the case where two or more rigid particles get close together [29]. According to a study by Crum [30], Gröschl [15] expressed a useful formula involving both compressibility and density effects of the secondary acoustic radiation force, taking into account the particle positions and their orientation in the acoustic field. For two particles with the same acoustic properties and same radii, *r*, this formula is given as:

(3) $$F_{\mathrm{se}}\left(y\right)=4\pi r^{6}\left\{\frac{{\left(\rho_{p}-\rho_{f}\right)}^{2}\left(3\cos^{2}\theta -1\right)}{6\rho_{f}d^{4}}v^{2}\left(y\right)-\frac{\omega^{2}\rho {\left(\kappa_{p}-\kappa_{f}\right)}^{2}}{9d^{2}}p^{2}\left(y\right)\right\}$$

where v(y) and p(y) are the acoustic velocity and pressure in the position *y* where the particles are located, respectively. Furthermore,  θ is the angle between the centerline connecting the two particles and wave propagation direction, ω is the angular frequency of acoustic wave, and *d* is center-to-center distance between the particles.

The first term of Equation (3) shows the dipole effect of the secondary acoustic force, which depends on θ and *d*^−4^. In this term, the density difference between the particle and the medium is important while it is independent of compressibility. It should be noted that in a standing wave, the dipole effect is dominant close to the pressure node where v(y) is maximal and p(y) is minimal. The second term, however, is the monopole effect which is not affected by θ. This means that the secondary acoustic force can be influential independent of the particle orientation in the acoustic field. In addition, the monopole part of the formula depends on the difference between the compressibility of the particles and the surrounding medium. For this reason, this term dominates the interaction force between bubbles having a much higher compressibility than solid particles or cells. Furthermore, the monopole term has a distance dependence decaying with *d*^−2^. In a standing wave, the monopole term is dominant close to the pressure antinode where p(y) is maximal and v(y) is minimal.

Equation (3) assumes acoustic interaction between particles of the same material and size. In this study, however, we investigate biological cells in acoustic interaction with silica particles. These objects have both different sizes and different material properties. For this reason, we have to extend the Gröschl model in Equation (3), in order to consider these differences. In a study by Apfel [28], the monopole part of the secondary acoustic radiation force between particles having different compressibilities and sizes can be expressed as:

(4) $$F_{\mathrm{mono}}=-\frac{\rho_{f}\kappa_{f}^{2}}{4\pi }{\left(\omega p\left(y\right)\right)}^{2}\left(1-\frac{\kappa_{p1}}{\kappa_{f}}\right)\left(1-\frac{\kappa_{p2}}{\kappa_{f}}\right)\frac{V_{1}V_{2}}{d^{2}}$$

where $V_{1}$ and $V_{2}$ are the volume of two particles and $\kappa_{p1}$ and $\kappa_{p2}$ are the compressibilities of the two particles. For the dipole part of the secondary acoustic radiation force between particles having different densities and sizes, Weiser and Apfel [29] came up with following formula:

(5) $$\begin{array}{c} F_{\mathrm{dipol}-r}=F_{0}\left(3\cos^{2}\theta -1\right)\;\\ F_{0}=\frac{2\pi \left(\rho_{f}-\rho_{p1}\right)\left(\rho_{f}-\rho_{p2}\right)r_{1}^{2}r_{2}^{2}}{3\rho_{f}d^{4}}v{\left(y\right)}^{2} \end{array}$$

where $F_{\mathrm{dipol}-r}$ is the radial component of the dipole part, and $\rho_{p1}$, $\rho_{p2}$, $r_{1}$, and $r_{2}$ are the densities and radii of the two different particles, respectively. In this study, we superposed the two equations (Equations (4) and (5)) in order to achieve a theoretical model for the total secondary acoustic radiation valid for two particles with different sizes and material properties in an arbitrary position in a one-dimensional acoustic standing wave. This model is used for calculating predicted acoustic interaction forces between a biological cell and a silica particle, and for comparison with our measured experimental interaction forces between these objects.

### 2.2. Non-Acoustic Forces

In addition to the secondary and primary acoustic radiation forces, non-acoustic forces such as lubrication forces and inertia forces can be of relevance when experimentally studying the dynamics of two nearby particles. Here, we briefly discuss the effect of each one of these forces.

#### 2.2.1. Lubrication Force

The lubrication force acts as a repulsive force and competes with the attraction force between two particles. In the case when two particles are attracted to each other due to the secondary acoustic force, the lubrication force reduces the effect of the acoustic force at very close distances. The lubrication force is proportional to the inverse of the distance between two particles and by decreasing the distance it increases. The effective lubrication force [31,32] becomes significant for surface-to-surface distances between two particles, $h_{0}$, where $0<h_{0}<\epsilon r$ and ϵ is a coefficient in the range of 0<ϵ≪1. Considering the radius of a red blood cell (RBC) (~3 to 4 μm), $h_{0}$ must be in range of about 300 nm for generating a significant lubrication force. As we cannot monitor the particle distance within this short range, therefore, we ignore the lubrication force when comparing our experimental results with theory.

#### 2.2.2. Hydrodynamic Force

In this study, we also neglect the effect of inertia forces due to the weaker particle acceleration which leads to inertia forces three orders of magnitude less than the secondary acoustic force in close proximity of two particles. In addition, we assume that the effect of hydrodynamic forces is negligible considering the very low Reynolds number of particles in motion in our experiments. As we describe in the following section, interaction between particles have been recorded under the condition where one particle is completely fixed (silica) and another particle (biological cell) is under motion close to the fixed particle. Therefore, we can also neglect hydrodynamic effects in the current study.

## 3. Materials and Methods

### 3.1. Experimental Apparatus

**Figure 1:**
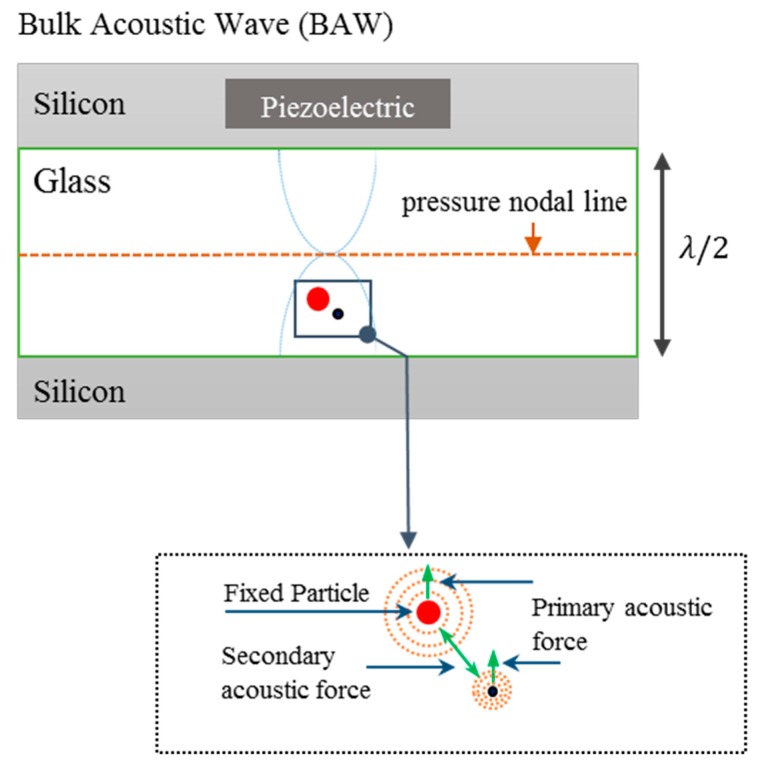
Top view of the experimental setup and method used to measure the acoustically generated motion of a red blood cell (RBC) or an MCF-7 cell (black particle) in close proximity to a fixed silica bead (red particle). A one-dimensional acoustic standing wave with a single pressure node in the center of the channel was produced while the fixed particle and moving cell are exposed to the ultrasonic wave resulting in both a primary and a secondary acoustic force.

The acoustofluidic device, previously described in [33], consists of a silicon-etched channel with cross-section 375 μm × 110 μm (width × height) designed to resonate at 2 MHz half-wavelength cross-sectional mode. For visual access, the top and bottom of the channel are covered with glass layers, of which one layer is compatible with high-resolution microscopy. The glass layers were anodically bonded onto the surfaces of the silicon wafer. The channel was actuated using a lead zirconium titanate (Pz-26, Ferroperm/Meggit A/S, Kvistgaard, Denmark) transducer with a 2 MHz serial resonance. In order to generate the acoustic field, the transducer was actuated with a signal generator (MFG-2120, Megatek, Taipei, Taiwan) with an output voltage of 10 V_pp_. The signal generator was connected to a digital oscilloscope (DSO-5070, Megatek, Taipei, Taiwan) to monitor the generated signal during the experiments. Particle motion during the experiments was monitored using an optical microscope (Leica-DM IL LED, Leica, Wetzlar, Germany) and a camera (Canon 1100-D, Canon, Tokyo, Japan). Syringes were used to inject particle samples, consisting of 20 µm silica beads, and either red blood cells (RBCs) or the breast cancer cell line MCF-7, into the channel. The flow in channel was controlled precisely with stop valves placed at both the inlet and the outlet sides of the channel. An overview of the experimental setup including the forces acting on the particle and cell are shown in Figure 1.

### 3.2. Fluid and Particle and Cell Properties

Silica microbeads with 20 μm diameters (Silica microparticles, Sigma-Aldrich, Merck KGaA, Darmstadt, Germany), and two different cell types, RBCs and MCF-7 cells, were used in the experiments. The properties of the particles, cells, and medium are listed in Table 1.

### 3.3. Experimental Procedure

Before starting each experiment, the following preparatory steps were performed subsequently: washing the channel by flushing deionized (DI) water, injecting a sample with 20 μm silica particles which were suspended in the pure DI water at the concentration of 10^4^ particles/mL, and resting the channel for 30 min in order to allow the silica particles to settle down completely. Since the silica particles were injected into the channel without using any detergent, they were intentionally attached to fixed positions at the channel bottom. The acoustic forces obtained in the experiments did not exceed the silica-channel adhesion forces, resulting in fixed positions of silica at any time. It should be noticed that after fixing the silica particles, the RBCs or MCF-7 cells were injected into the channel at a cell concentration of about 10^5^ cells/mL. In order to find the secondary acoustic force between a silica particle and an RBC or a MCF-7 cell, one or two cells were initially positioned in the proximity of the 20 μm silica particles. Cell positioning was controlled manually by using a syringe and a stop valve. The silica particles with desired acoustic properties were chosen carefully to optimize the effect of the secondary acoustic force and compensate for the material properties of biological cells causing weak acoustic contrast when suspended in water. After this initial procedure, the channel was exposed to the resonant acoustic field at 2 MHz. The trajectories of the cells were analyzed by the use of the camera recording images at 25 frames per second (fps). The trajectories were then used to extract the cell velocities in the horizontal plane by the use of a particle tracking method. By this method, location and speed of the particles can be easily determined and used in further calculations. Consequently, the total force acting on cells could be evaluated. By extracting this data we used Equations (4) and (5), and compared the theory with experimental data. All videos were analyzed using Tracker, an open source particle tracing software [36].

## 4. Results and Discussion

### 4.1. Interparticle Force Estimation

In order to investigate the secondary acoustic radiation force, two different series of experiments were implemented using fixed silica beads and motile RBCs or MCF-7 cells. To estimate the acoustic radiation forces acting on the particles and cells, we first need to measure the acoustic energy density in the channel. By considering that we have no fluid flow in the channel during the experiments, we can balance the acoustic force with the Stokes drag force:

(6) $$F_{\mathrm{drag}}=6\pi \eta vr_{p}$$

where η is dynamic viscosity, v is the velocity of particle relative to the surrounding medium, and $r_{p}$ is cell radius. Knowing $r_{p}$ and v by the use of the particle tracking method we can find $F_{\mathrm{drag}}$ in each video frame. By using this force balancing procedure and considering that the standing wave field in our device is purely one-dimensional, the primary and secondary acoustic radiation forces can be decoupled and evaluated in the wave propagation direction (primary force) and perpendicular direction to the wave propagation (secondary force), respectively. Thus, the perpendicular force component used for measuring the secondary radiation force was compared with the extended Gröschl model [15], Equations (4) and (5).

### 4.2. Energy Density in the Channel

To find the energy density in the channel, Stokes drag formula combined with Gorkov’s equation (Equations (1) and (2)) were used. The energy density was evaluated based on tracking non-fixed silica beads using the particle tracking method [37] in one-dimensional acoustic field at the applied actuation voltage 10 V_pp_. In this condition the energy density of the channel was measured to be 0.5 to 1.3 J/m^3^. During this procedure, we did not notice any particle motion in the direction perpendicular to the standing wave.

### 4.3. Effect of the Secondary Acoustic Force on Cell Movement

**Figure 2:**
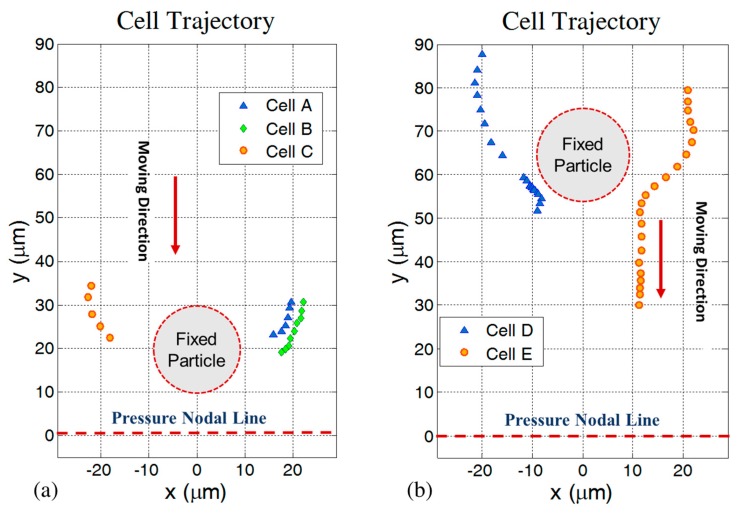
Trajectories of RBCs due to a combination of the primary and the secondary acoustic forces. (**a**) Three different experiments with the silica particle fixed at a vertical distance of 20 µm from the pressure nodal line, the latter set to *y* = 0. In the A, B, and C cases, the cells were trapped at the silica particle and stacked. Note that the shortest distance between the positions of the cells and the boundary of the fixed particle corresponds to the radius of the RBCs. (**b**) Two different experiments with the silica particle fixed at a vertical distance of 65 µm from the pressure nodal line. In both cases, cells D and E were separated from the fixed silica particle following a smooth contact.

To investigate the motion behavior of cells in close proximity of fixed silica particles during acoustic exposure, different experiments were performed. In Figure 2 the trajectories of RBCs in the horizontal *x*-*y* plane initially located in the proximity of fixed silica particles are shown from five repetitions of the experiment. In Figure 2a, the position of silica particle is constant during three different tests. To compare RBCs trajectories with each other we show them in the same picture. Figure 2b also shows two cases with the same silica particle position. In both Figure 2a,b, the pressure node is in the middle of the channel and located below (line *y* = 0) the fixed silica particle, while the RBCs start moving from the upper side. High deflection in the RBCs’ trajectory (along the *x* direction) in the vicinity of fixed particle can be seen clearly while there is no contact between cells and the silica particle. In almost all cases, the center-to-center distances between cells and silica particles are about 20 to 25 µm and in three out of five cases the secondary acoustic force is strong enough to stick RBCs to the fixed silica particle, near *θ* = 90° (where *θ* is the angle between centerline of cell-silica particle and wave propagation direction in the one-dimensional standing wave). In our case, results show that when the silica particle is located in between the pressure nodal line and up to about 50 µm from this line, all the RBCs (*N* = 4) stick to the silica particle. Farther away from this zone the ability of trapping RBCs gradually gets weaker. In the region close to the channel wall we observed pathway deflection without sticking cells. In the region far from the pressure nodal line the monopole term of the secondary acoustic force is dominating and the probability of sticking of a RBC to a silica particle is minimum as compared with the case when the silica particle is located close to the pressure nodal line where the dipole terms of secondary acoustic force is dominating. Additionally, it should be noticed that the primary acoustic radiation force close to the pressure node reduces dramatically and this causes a perfect condition for secondary acoustic radiation force-dependent cell trapping to silica particles. Furthermore, as shown in the figure cells with the same initial position undergo the same deflection behavior, which is quantitatively in agreement with the Gröschl theory and also in agreement with the experimental reproducibility obtained in [27]. It can be seen in the figure that the pathways of the RBCs are almost straightforward along the *y*-axis before and after they get close to the fixed silica particle.

**Figure 3:**
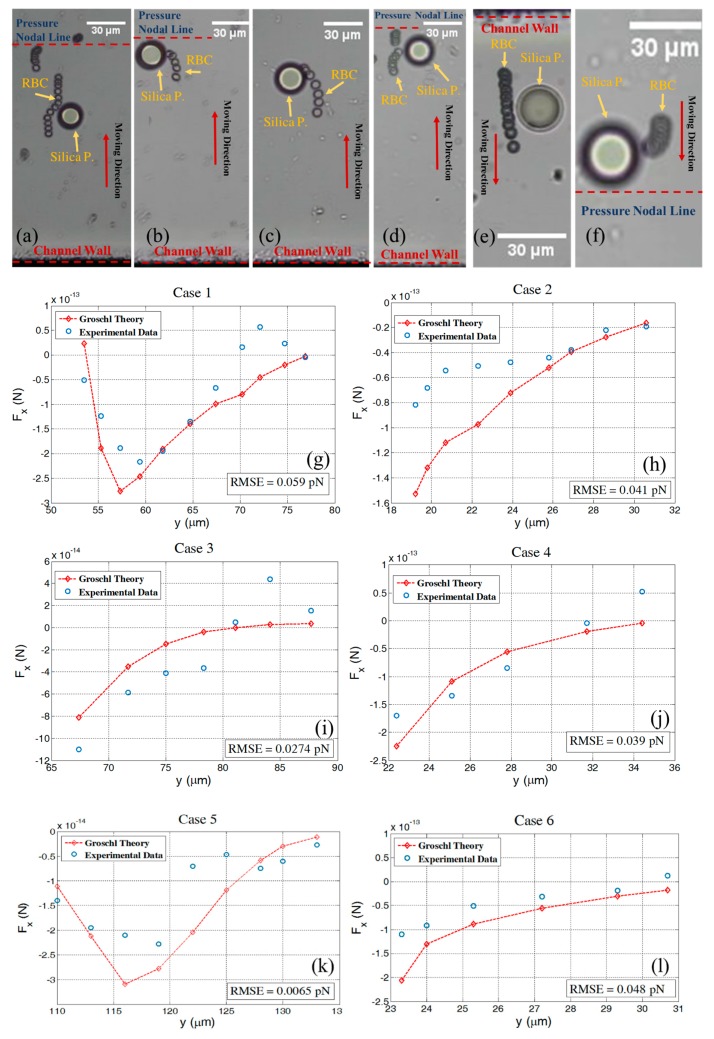
Time series of RBC pathways (**a**–**f**), cases 1 to 6, respectively, and experimental and theoretical comparison of the secondary acoustic force while RBCs pass by a fixed 20 µm silica particle based on the extended Gröschl model (**g**–**l**). Standard deviations of the residuals were shown for each case (root mean square error, RMSE). In (**c**,**e**), the pressure nodal line is outside of the picture. In (**f**) the channel wall is outside of picture. The center of the fixed silica particle in (**g**,**i**) is located at 65 µm, in (**h**,**j**,**l**) at 20 µm, and (**k**) at 128 µm from the pressure nodal line (the latter set to *y* = 0).

In the following six different experiments, we investigated in more detail the RBC and silica interaction. Here we analyze time series of cell motion for estimating an experimental secondary acoustic force, and we compare this with the extended Gröschl theory, see Figure 3. The comparison was performed for different initial positions of RBCs in wave propagation direction while the origin of the coordinate system is located on the pressure nodal line. In all cases, when the RBCs responded to the acoustic wave, they moved in the wave propagation direction towards the pressure nodal plane until they appeared in close proximity to the fixed silica particle. In this position, an incurvature of the RBCs’ pathways was observed which lead to either trapping or just a deflection based on the strength of the secondary acoustic force relative to the primary acoustic force. As soon as the moving RBCs passed the fixed silica particle they continued straightforwardly towards the pressure nodal plane, cf. Figure 3a,e. As shown in all six experiments there is good agreement between experimental and theoretical data. In Figure 3a,e, we can see that a moving RBC does not stick to a fixed silica particle, instead it shows a distinctive deflection when passing by a fixed particle. This behavior can also be seen in the corresponding force graphs, Figure 3g,k, where the secondary acoustic force magnitudes are at maximum when an RBC and a particle are at the same *y* level. Exactly after this, it can be seen that the magnitude of the secondary acoustic force reduces rapidly. In Figure 3i a few data points show a different trend as compared with the theoretical data. This behavior can be referred to a small fluctuation in the acoustic wave or instability of the fluid flow which we assume is zero. In addition, in Figure 3g the same behavior can be seen; in this case the trajectory behavior occurs very close to the separation point of the cell from the silica particle. In this condition, lubrication force can become significant and influence the trajectory of the cell pathway.

**Figure 4:**
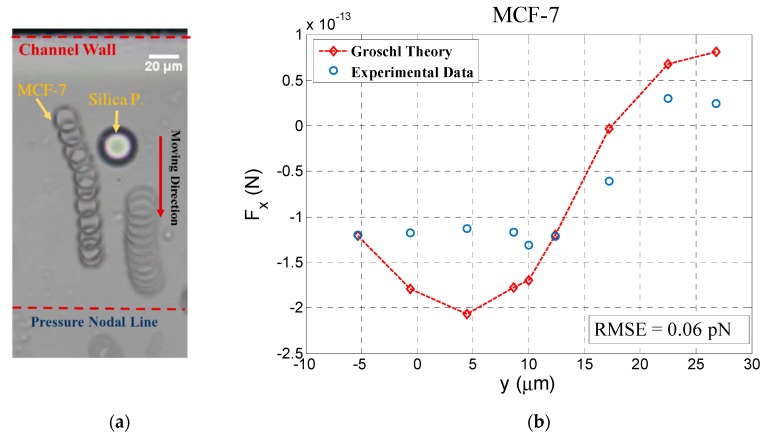
Time series of MCF-7 pathways while moving close to the fixed silica particle (**a**), and comparison of experimental data of the secondary acoustic force between a MCF-7 cell and silica particle with the extended Gröschl model (**b**). Here, the center of the fixed silica particle is located at 108 µm from the pressure nodal line.

Considering that most applications of acoustofluidic systems today are related to the separation or manipulation of cancer cells which usually have larger sizes than RBCs, we added an experiment using the cancer cell line MCF-7 in order to investigate the impact of secondary acoustic force for such applications. Using the same procedure as for RBCs, MCF-7 cells were investigated and significant deflection in cell pathway was observed. The trajectory and the secondary acoustic force obtained from experimental data were compared with theory. Figure 4 depicts this result, which shows a good agreement between experimental and theoretical data. In this figure the negative values on the *x* axis relate to the cell position before reaching the silica bead and the positive values refer to the cell position when it has passed the silica bead. As shown in the time series picture, after passing the silica bead, the MCF-7 has almost a straightforward pathway in the wave propagation direction towards the pressure node. In this figure we also see the MCF-7 pathway deflection in the proximity of the fixed silica bead. Theoretical data shows the rapid changes in secondary acoustic force while a MCF-7 cell passes by a silica particle.

### 4.4. Effect of Cell Concentration on the Secondary Acoustic Force

On the basis of our observations of the secondary acoustic force on interparticle behavior in a microchannel, we conclude that such acoustofluidic systems with high particle and cell concentrations can experience interparticle and cell forces to the level which can cause high purity reduction in acoustophoresis-based separation methods. Here, we investigate a basic condition where we assume that there is a uniform cell and particle distribution in the medium. In this situation different concentrations of MCF-7 cells in one-dimensional standing wave have been studied theoretically. The aim of this section is to answer the question of whether secondary acoustic forces can cause significant changes at certain concentrations of particles and cells in standard acoustophoresis applications. To address this question, cell concentrations up to 7 × 10^6^ cells/mL were considered. In a uniform particle distribution, the distance to each particle from its neighbor can be evaluated by the particle density (*N*/*V*) where, *V* is the volume of medium and *N* is the total number of particles and cells that exists in the medium. By the use of the following formula we can define an equal radius according to:

(7) $$r_{\mathrm{equal}}={\left(\frac{3V}{4\;\pi N}\right)}^{1/3}$$

**Figure 5:**
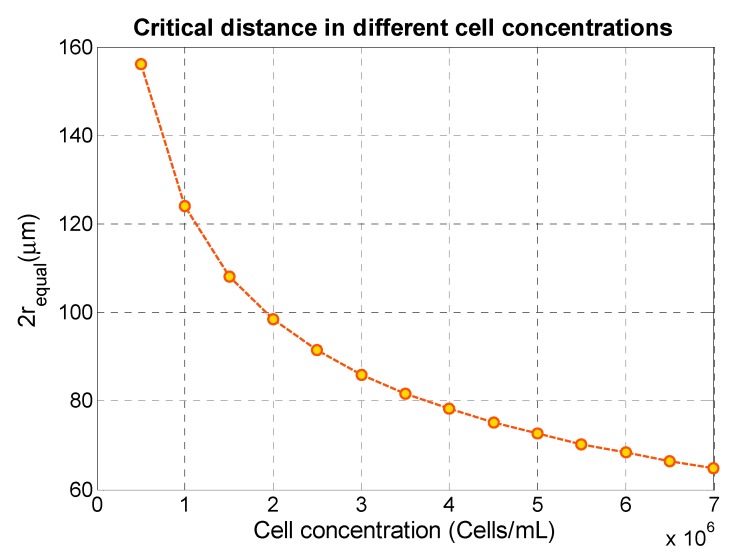
Center-to-center distance between two neighboring particles in a uniform distribution of MCF-7 as a function of different cell concentrations.

Here, the center-to-center distance between two neighboring particles in a uniform distribution is equal to 2 $r_{\mathrm{Equal}}$. If the particle concentration is chosen in the range that results in particle–particle distances larger than a critical distance, we can expect less interaction between cells or particles due to the secondary acoustic force. Considering the cell line MCF-7 with a typical diameter of 20 µm, we found by analyzing our experimental data that this critical distance is about 80 µm for MCF-7 cells and about 35 µm for RBCs, in proximity of a silica particle. By calculating $2r_{\mathrm{equal}}$ for different cell concentrations and comparing this distance with the critical distance, we can estimate the degree of cell–particle interaction due to acoustic forces. Figure 5 shows as an example the relation between cell concentration and $2r_{\mathrm{equal}}$ for MCF-7 cells. As shown, at MCF-7 cell concentrations larger than 3.5 × 10^6^ cells/mL, $2r_{\mathrm{equal}}$ becomes smaller than the critical distance 80 µm and we can expect that the secondary acoustic force would be of relevance for the acoustophoretic function. Considering a non-uniform cell distribution resulting in a locally smaller $2r_{\mathrm{equal}}$, the secondary acoustic force could be of even larger relevance.

Here, we used experimental data to determine the critical distance. However, to design an acoustophoretic microchannel, the user can also combine the extended Gröschl model with the Gorkov model to calculate the secondary and primary acoustic forces in various locations in a standing wave, respectively. In a certain location, based on the relative magnitude of the secondary acoustic force, $F_{\sec\mathrm{ondary}}/F_{\mathrm{primary}}$, the critical distance can be predicted. Typically, for the force ratio, $F_{\sec\mathrm{ondary}}/F_{\mathrm{primary}}$, larger than 0.1, we observe particle and cell pathway deflections, and for a force ratio close to 1 or more, we observe particle and cell sticking. Obviously, the critical distance would decrease in the case of two biological cells interacting due to weaker acoustical properties of cells, and the distance would increase in the case of interaction between two silica particles in the same condition. Thus, we expect a lower critical distance for pairs of cells such as for an MCF-7 cell and an RBC.

## 5. Conclusions

In this study, we have investigated the secondary acoustic radiation force acting between biological cells, including RBCs and MCF-7 cells, and silica particles in an ultrasonic standing wave. The force measurements were performed in an arbitrary position in a half wavelength wide microchannel. Results show good agreement between experimental and theoretical data. Our results indicate that, in the acoustic field, cells in close proximity of silica particles can cause cell pathway deflection in the transversal direction relative to the wave propagation direction. We also found that the secondary acoustic radiation force can overcome the primary acoustic radiation force, resulting in RBCs being trapped by the silica particles at positions in between a pressure node and a pressure anti-node. This new trapping principle was observed for the RBCs, but not for the larger MCF-7 cells. This finding could open up a new trapping method using secondary acoustic radiation forces for isolation of biological cells. In addition, measuring the secondary acoustic force could also be utilized as a new method in mechanical and acoustical characterization of biological cells. Furthermore, by identifying a critical distance between cells where the secondary acoustic radiation force becomes significant, we can estimate a corresponding critical cell concentration where secondary acoustic radiation forces are significant and must be considered as an important parameter when designing acoustophoresis devices and methods. To our knowledge this is the first experimental study of secondary acoustic radiation forces involving biological cells in microchannel acoustophoresis.

## Figures and Tables

**Table 1:** Acoustic properties of the fluid and microparticles and cells.

Material	Density [kg/m^3^]	Speed of Sound [m/s]	Radius [µm]
Water	998	1480	-
Silica beads	2331	8490	10
RBC	1139 [34]	1680 [34]	3.1 *
MCF-7	1068 [35]	1530 [35]	7.7

* Standard deviation 0.21 µm (*N* = 10).

## References

[1] King L.V. (1934). On the acoustic radiation pressure on spheres. Proceedings of the Royal Society of London. Series A Math. Phys. Sci..

[2] Gor’kov L.P. (1962). On the forces acting on a small particle in an acoustical field in an ideal fluid. Sov. Phys. Dokl..

[3] Yosioka K., Kawasima Y. (1955). Acoustic radiation pressure on a compressible sphere. Acustica.

[4] Hasegawa T. (1969). Acoustic-Radiation Force on a Solid Elastic Sphere. J. Acoust. Soc. Am..

[5] Doinikov A.A. (1997). Acoustic radiation force on a spherical particle in a viscous heat-conducting fluid. I. General formula. J. Acoust. Soc. Am..

[6] Doinikov A.A. (1997). Acoustic radiation force on a spherical particle in a viscous heat-conducting fluid. III. Force on a liquid drop. J. Acoust. Soc. Am..

[7] Bruus H., Dual J., Hawkes J., Hill M., Laurell T., Nilsson J., Radel S., Sadhal S., Wiklund M. (2011). Forthcoming Lab on a Chip tutorial series on acoustofluidics: Acoustofluidics-exploiting ultrasonic standing wave forces and acoustic streaming in microfluidic systems for cell and particle manipulation. Lab Chip.

[8] Lenshof A., Magnusson C., Laurell T. (2012). Acoustofluidics 8: Applications of acoustophoresis in continuous flow microsystems. Lab Chip.

[9] Meng L., Cai F., Jin Q., Niu L., Jiang C., Wang Z., Wu J., Zheng H. (2011). Acoustic aligning and trapping of microbubbles in an enclosed PDMS microfluidic device. Sens. Actuators B Chem..

[10] Roux-Marchand T., Beyssen D., Sarry F., Elmazria O. (2015). Rayleigh surface acoustic wave as an efficient heating system for biological reactions: Investigation of microdroplet temperature uniformity. IEEE Trans Ultrason. Ferroelectr. Freq. Control..

[11] Muller P.B., Bruus H. (2015). Theoretical study of time-dependent, ultrasound-induced acoustic streaming in microchannels. Phys. Rev. E Stat. Nonlin. Soft Matter Phys..

[12] Lighthill S.J. (1978). Acoustic streaming. J. Sound Vib..

[13] Wiklund M., Green R., Ohlin M. (2012). Acoustofluidics 14: Applications of acoustic streaming in microfluidic devices. Lab Chip.

[14] Settnes M., Bruus H. (2012). Forces acting on a small particle in an acoustical field in a viscous fluid. Phys. Rev. E.

[15] Gröschl M. (1998). Ultrasonic Separation of Suspended Particles-Part I: Fundamentals. Acustica.

[16] Bjerknes V.F.K. (1906). Fields of Force.

[17] Crum L.A. (1975). Bjerknes forces on bubbles in a stationary sound field. J. Acoust. Soc. Am..

[18] Doinikov A.A. (1999). Bjerknes forces between two bubbles in a viscous fluid. J. Acoust. Soc. Am..

[19] Zheng X., Apfel R.E. (1995). Acoustic interaction forces between two fluid spheres in an acoustic field. J. Acoust. Soc. Am..

[20] Garcia-Sabaté A., Castro A., Hoyos M., González-Cinca R. (2014). Experimental study on inter-particle acoustic forces. J. Acoust. Soc. Am..

[21] Silva G.T., Bruus H. (2014). Acoustic interaction forces between small particles in an ideal fluid. Phys. Rev. E.

[22] Sepehrirahnama S., Lim K.M., Chau F.S. (2015). Numerical study of interparticle radiation force acting on rigid spheres in a standing wave. J. Acoust. Soc. Am..

[23] Baasch T., Leibacher I., Dual J. (2017). Multibody dynamics in acoustophoresis. J. Acoust. Soc. Am..

[24] Wang S., Allen J.S., Ardekani A.M. (2017). Unsteady particle motion in an acoustic standing wave field. Eur. J. Comput. Mech..

[25] Habibi R., Devendran C., Neild A. (2017). Trapping and patterning of large particles and cells in a 1D ultrasonic standing wave. Lab Chip.

[26] Mohapatra A.R., Sepehrirahnama S., Lim K. (2018). Experimental measurement of interparticle acoustic radiation force in the Rayleigh limit. Phys. Rev. E.

[27] Saeidi D., Saghafian M., Javanmard S., Hammarstrom B., Wiklund M. (2019). Acoustic dipole and monopole effects in solid particle interaction dynamics during acoustophoresis. J. Acoust. Soc. Am..

[28] Apfel R.E. (1988). Acoustically induced square law forces and some speculations about gravitation. Am. J. Phys..

[29] Weiser M.A., Apfel R.E., Neppiras E.A. (1984). Interparticle forces on red cells in a standing wave field. Acta Acust. United Acust..

[30] Crum L.A. (1971). Acoustic force on a liquid droplet in an acoustic stationary wave. J. Acoust. Soc. Am..

[31] Vázquez-Quesada A., Ellero M. (2016). Analytical solution for the lubrication force between two spheres in a bi-viscous fluid. Phys. Fluids.

[32] Lambert B., Weynans L., Bergmann M. (2018). Local lubrication model for spherical particles within incompressible Navier-Stokes flows. Phy. Rev. E.

[33] Manneberg O., Svennebring J., Hertz H.M., Wiklund M. (2008). Wedge transducer design for two-dimensional ultrasonic manipulation in a microfluidic chip. J. Micromech. Microeng..

[34] Mishra P., Hill M., Glynne-Jones P. (2014). Deformation of red blood cells using acoustic radiation forces. Biomicrofluidics.

[35] Hartono D., Liu Y., Tan P.L., Then X.Y.S., Yung L.-Y.L., Lim K.-M. (2011). On-chip measurements of cell compressibility via acoustic radiation. Lab Chip.

[36] Brown D. Tracker Free Video Analysis and Modeling Tool for Physics Education. Edition 4.11.0, Open Source Physics compadre. https://arxiv.org/abs/1308.2614.

[37] Barnkob R., Augustsson P., Laurell T., Bruus H. (2010). Measuring the local pressure amplitude in microchannel acoustophoresis. Lab Chip.

